# Impaired SERPIN–Protease Balance in the Peripheral Lungs of Stable COPD Patients

**DOI:** 10.3390/ijms26072832

**Published:** 2025-03-21

**Authors:** Antonino Di Stefano, Francesco Nucera, Umberto Rosani, Paola Brun, Isabella Gnemmi, Mauro Maniscalco, Silvestro Ennio D’Anna, Andrea Leonardi, Vitina Carriero, Francesca Bertolini, Josè Freni, Antonio Ieni, Sebastiano Gangemi, Paolo Ruggeri, Fabio Luigi Massimo Ricciardolo

**Affiliations:** 1 Divisione di Pneumologia e Laboratorio di Citoimmunopatologia dell’Apparato Cardio Respiratorio, Istituti Linici Scientifici Maugeri, IRCCS, Respiratory Rehabilitation Unit of Gattico-Veruno, 28013 Novara, Italy; isabella.gnemmi@icsmaugeri.it; 2 Pneumologia, Dipartimento di Scienze Biomediche, Odontoiatriche e delle Immagini Morfologiche e Funzionali (BIOMORF), Università degli Studi di Messina, Piazza Pugliatti 1, 98122 Messina, Italy; francesco.nucera@unime.it (F.N.); jose.freni@unime.it (J.F.); paolo.ruggeri@unime.it (P.R.); 3 Department of Biology, University of Padova, Via U. Bassi 58/b, 35121 Padova, Italy; umberto.rosani@unipd.it; 4 Histology Unit, Department of Molecular Medicine, University of Padova, 35121 Padova, Italy; paola.brun@unipd.it; 5 Divisione di Pneumologia, Istituti Clinici Scientifici Maugeri, IRCCS, Telese, 82037 Benevento, Italy; mauro.maniscalco@icsmaugeri.it (M.M.); silvestro.danna@icsmaugeri.it (S.E.D.); 6 Ophthalmology Unit, Department of Neuroscience, University of Padova, 35121 Padova, Italy; andrea.leonardi@unipd.it; 7 Severe Asthma, Rare Lung Disease and Respiratory Pathophysiology Unit, Department of Clinical and Biological Sciences, University of Turin, San Luigi Gonzaga University Hospital, Orbassano, 10043 Turin, Italy; vitina.carriero@unito.it (V.C.); francesca.bertolini@unito.it (F.B.); 8 Department of Human Pathology in Adult and Developmental Age ‘Gaetano Barresi’, Section of Pathology, University of Messina, 98122 Messina, Italy; antonio.ieni@unime.it; 9 Operative Unit of Allergy and Clinical Immunology, Department of Clinical and Experimental Medicine, University of Messina, 98125 Messina, Italy; sebastiano.gangemi@unime.it

**Keywords:** SERPIN signaling, pulmonary rehabilitation, airway inflammation, stem cells, endothelial cells, bronchiolar epithelial cells, pulmonary emphysema

## Abstract

The protease–antiprotease balance is involved in many biological processes, including blood coagulation, tissue remodeling, inflammation and immune responses. The aim of this study is to determine the balance between SERPINs and some related proteases in the lungs of stable COPD patients. In this cross-sectional study, the expression and localization of human SERPINs (anti-proteases) and some related proteases were measured in the lung parenchyma of mild-moderate COPD (MCOPD, n = 13) patients, control smokers (CS, n = 14) and control nonsmokers (CNS, n = 12) using transcriptome analysis, immunohistochemistry, and ELISA tests. Peripheral lung transcriptomic data showed increased mRNA levels of tissue plasminogen activator (tPA), cathepsin-L and caspase-1 as well as increased SERPINs A6, B3, B5, B11, B13 in the COPD group compared to the CNS group. At the protein level, IHC analysis showed that tPA and cathepsin-L increased in the bronchiolar epithelium and alveolar septa of the CS and COPD groups compared to the CNS group, as well as SERPINB5 and B13 in the alveolar macrophages and alveolar septa of the CS and COPD groups compared to the CNS group. SERPINA6 was shown to be decreased in the bronchiolar epithelium, bronchiolar lamina propria, and alveolar septa of the CS and COPD groups compared to the CNS group and was positively correlated with lung function. SERPINB3 was decreased in the alveolar septa of the CS group compared to the CNS group. The ELISA tests showed that in the total lung extracts, decreased levels of SERPINA6 and increased caspase-1 were shown in the COPD group compared to the CNS or both control groups, respectively. These data show an imbalance, at the protein level, of SERPINs and some related proteases in the lungs of the CS and stable COPD groups. These alterations may play a role in damaging the lung parenchyma of susceptible COPD patients.

## 1. Introduction

The analysis of SERPINs in patients with COPD is fragmentary and at least in part, contradictory.

SERPINs are mainly protease inhibitors [1]. In humans, 4/5 of the 37 identified SERPINs are inhibitory in function and are involved in many biological processes, including tissue remodeling, blood coagulation cascade, thrombosis, and inflammatory responses [1]. Non-inhibitory functions have also been reported, which include hormone transport (SERPINA6, SERPINA7), blood pressure regulation (SERPINA8), and tumor suppression (SERPINB5) [1]. SERPINA1 (AAT) deficiency and its polymerization may cause emphysema and cirrhosis [2]. Most SERPINs, separated into nine clades (A–I) in humans, are extracellular molecules, while intracellular ones, usually belonging to clade B, tend to be tissue-specific and participate in cellular events [1]. Considering functionally the selected SERPINs and proteases included in this study, SERPINA3 (alpha-1-anti-chymotrypsin) inhibits chymotrypsin, mast cell chymases, kallikreins 2 and 3 and cathepsin G [3]; SERPINA6 (corticosteroid-binding globulin), a non-inhibitory serine protease inhibitor, is the primary cortisol-binding protein involved in the regulation of acute, severe, and chronic inflammation [4]; SERPINB2, known as plasminogen activator inhibitor-2 (PAI-2) is a major intracellular inhibitor of tissue and urokinase plasminogen activator (tPA, uPA) [5]; SERPINB3 inhibits cysteine proteases, cathepsins K, L, S and papain [6,7], promotes epithelial cell proliferation, and inhibits inflammation. An antiangiogenic activity for SERPINB5 has been reported; however, its functions need to be better explored [1,8]. SERPINB11 is a non-inhibitory serine protease for which functions related to host-pathogen interactions to fight infectious diseases have been proposed [9]. SERPINB13 inhibits cathepsins K and L inhibiting lysosomal cysteine proteinases [10]. The activity of thrombin in the vasculature is controlled by SERPINC1 (antithrombin) and SERPIND1 (heparin cofactor II). In exacerbated COPD a reduced SERPINC1 activity [11], associated with coagulation activation and hypoxia [12] was reported, whereas the levels and actions of SERPIND1 were not reported [13] in COPD. SPINK1 (serine peptidase inhibitor Kazal type 1) is a trypsin inhibitor with diverse physiological functions such as promoting cell proliferation and migration [14] while preventing cell apoptosis. A direct relationship between SPINK1 with COPD has not been reported. PLAUR (UPAR, urokinase-type plasminogen activator receptor) contributes to plasminogen activation, chemotaxis, cell adhesion; and immune cell activation [15]; it is increased in COPD and correlated with the severity of the disease [16]. Tissue-plasminogen activator (tPA) is involved in dissolving blood clots [17] and, after plasmin activation, may have pro-angiogenic [18] activity. Cathepsin K and L are lysosomal cysteine proteases involved in protein degradation [19]. Elastinolytic activity “in vivo” has been reported for cysteine cathepsins involved in atherosclerosis and pulmonary emphysema [20]. Cathepsins have been shown to modulate innate immunity by degrading antimicrobial peptides [21] and to inactivate secretory leuco-protease inhibitor (SLPI) and its anti-neutrophil elastase capacity [22]. Cathepsin L activity and its mRNA levels were increased in the alveolar macrophages of smokers compared to nonsmokers [23]. Conflicting results are reported for inflammasome activation via the “canonical pathway” involving caspase 1 functions in stable COPD patients [24,25], which were found to be upregulated by some authors [24] or not changed by others [25].

A progressive reduction in the numbers of peripheral airways has been reported in patients with COPD [26,27]. These molecular alterations are also present in smokers with near-normal lung function [26,27]. Proteases have been directly implicated in these structural alterations developing in COPD of increasing severity and in emphysema [1,27]. We aimed to study the balance between SERPINs and some related proteases in the peripheral airways and lung parenchyma of stable mild-moderate COPD compared to control smokers and nonsmoking subjects.

## 2. Results

### 2.1. Clinical Characteristics of Subjects Providing Lung Parenchyma

We obtained and studied peripheral lung samples from the lung resections of 39 subjects, whose characteristics are shown in Table 1.

Thirteen of these had COPD, while the other 26 had normal lung function. Fourteen of the control subjects were smokers.

### 2.2. Gene Expression Level of SERPIN-Signaling Molecules in Lung Parenchyma

**Figure 1:**
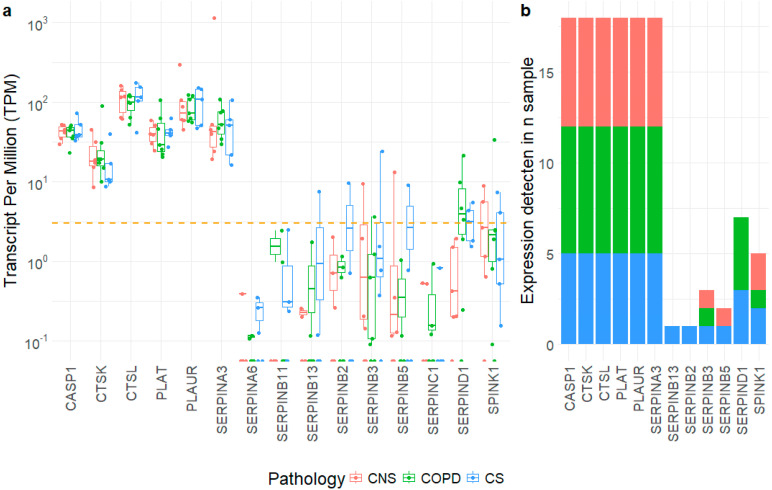
Expression levels of 15 selected genes obtained from lung parenchyma of control nonsmokers (CNS, n = 6), control smokers (CS, n = 5) and patients with chronic obstructive pulmonary disease (COPD, n = 7). The box plot shows the median and the distribution of expression values per gene reported as transcript per millions (TPMs): (**a**) the orange dotted line shows the lower detection limit arbitrary set to 3 TPMs; and (**b**) the number of samples with detectable expression levels for the same genes, divided by conditions. The color codes are as follows: red for CNS; green for COPD; and blue for CS samples.

We examined whole-transcriptome RNA-sequencing expression datasets obtained from the lung parenchyma (CNS, n = 6; COPD, n = 7, CS, n = 5) used for immunohistochemical analysis. After a preliminary analysis of 57 molecules (Table S2, Supplementary Materials), we extracted the expression levels of 15 selected SERPIN-signaling molecules (Figure 1a).

Overall, we observed two gene expression conditions, with the first including genes with higher expression levels such as caspase 1, cathepsins, tissue-type plasminogen activator (PLAT), urokinase plasminogen activator surface receptor (PLAUR) and a single SERPIN gene (SERPINA3). These genes are characterized by expression levels ranging generally over 20 TPMs and reaching almost 300 TPMs as SERPINA3 in one CNS sample. Despite their considerable expression levels, which appeared detectable in all the sample types (Figure 1b), these genes were poorly regulated among the tested conditions. A second group of genes included most of the considered SERPINs and serine protease inhibitor Kazal-type 1 and are characterized by lower expression levels, generally below 10 TPMs, with a relevant number of samples showing no detected expression levels (TPMs < 3), particularly considering the COPD and CNS samples (Figure 1b).

### 2.3. Immunohistochemistry for SERPIN-Signaling Proteins in the Peripheral Airways and Lung Parenchyma

The minimum internal diameter of the bronchioles studied, ranging between 250 to 450 microns, showed that peripheral bronchioles between eleventh and sixteenth generation were analyzed in all groups of patients and control subjects. In the three groups of subjects, a similar number of peripheral bronchioles, between 6 to 11 for each subject, were studied, similarly to a previous report. We observed that the bronchiolar number was tendentially lower in COPD patients (https://doi.org/10.3390/ijms25063287) [26,27].

We examined bronchiolar epithelial cells, bronchiolar lamina propria, alveolar macrophages, alveolar septa, and lung vessels.

In bronchiolar epithelium, decreased levels of SERPINA6 immunopositivity was observed in CS compared to CNS (Table 2).

**Figure 2:**
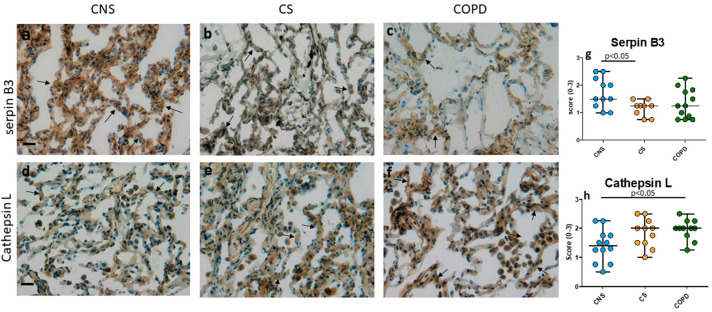
Photomicrographs showing alveolar septa from: (**a**,**d**) control nonsmokers; (**b**,**e**) control smokers, and (**c**,**f**) mild-moderate stable COPD patients immunostained for the identification of SERPINB3+ cells (**a**–**c**) and cathepsin L+ cells (**d**–**f**). Results are representative of those from 12 nonsmokers, 14 smokers with normal lung function and 13 with mild-moderate stable COPD. SERPINB3 is tendentially decreased in the alveolar septa of COPD patients compared to CNS. Cathepsin L is increased in the alveolar septa of COPD patients compared to CNS. Arrows indicate some immunopositive alveoli. Panels (**g**,**h**) show the quantitative analyses performed in lamina propria for immune expression of SERPINB3 (**g**) and cathepsin L (**h**). CNS, control nonsmokers; CS, control smokers with normal lung function, COPD, chronic obstructive pulmonary disease. Bar = 20 micron.

In COPD, this value tended to be decreased as well. SERPINB5 increased in CS compared to CNS and COPD (Table 2). TPA increased in CS and COPD compared to CNS (Table 2). In bronchiolar lamina propria, SERPINA6 levels decreased in CS and COPD compared to CNS (Table 2). SERPINB13 increased in CS and COPD compared to CNS (Table 2). Caspase 1 decreased in CS compared to CNS (Table 2). In alveolar macrophages, SERPINB5 increased in CS compared to CNS (Table 2). SERPINB13 was increased in CS and COPD compared to CNS (Table 2). PLAUR (UPAR) increased in CS compared to CNS and COPD (Table 2). Cathepsin L tended to be increased in CS and COPD compared to CNS (Table 2). In alveolar septa, SERPINB3 tended to be decreased in CS compared to CNS (Table 2, Figure 2).

SERPINB5 and B13 was increased in CS and COPD compared to CNS (Table 2). SERPINB11 was decreased in CS compared to CNS and COPD (Table 2). Cathepsin L was increased in COPD compared to CNS (Table 2, Figure 2). Caspase 1 was decreased in CS compared to CNS (Table 2). In lung vessel endothelium, SERPINA6 tended to be decreased in CS compared to CNS (Table 2). SERPINB11 was decreased in CS compared to CNS and COPD (Table 2). SERPINB13 and tPA (endothelium and smooth muscle) was increased in CS and COPD compared to CNS (Table 2). Endothelial caspase 1 was decreased in CS and COPD compared to CNS (Table 2). In all lung compartments studied, the least expressed SERPINs were SERPINA3, SERPINB2 (PAI-2) and SERPIND1 (Table 2).

### 2.4. ELISA Tests for SERPIN-Signaling Proteins in Homogenized Peripheral Lung Tissue

**Figure 3:**
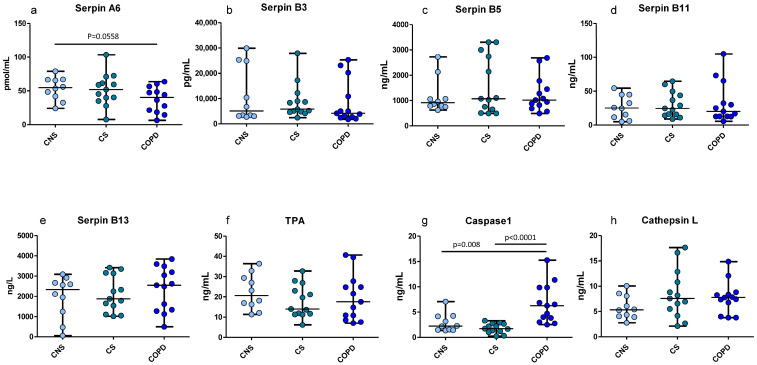
ELISA tests performed on frozen lung tissue homogenates of patients with COPD (n = 13), CS (n = 14) and CNS (n = 12) for quantitation of SERPINs A6, B3, B5, B11, B13, tPA, caspase1 and cathepsin L: (**a**) SERPINA6 tendentially decreased in COPD compared to CNS; (**g**) caspase 1 increased in COPD compared to CNS and CS; and (**b**–**f**,**h**) no other significant differences were observed. The Mann–Whitney test was applied for comparison between groups. CNS, control nonsmokers; CS, control smokers with normal lung function, COPD, chronic obstructive pulmonary disease.

As shown in Figure 3, we found significant differences in the concentration of SERPINA6, which was decreased in the total lung protein extracts of mild moderate COPD compared to CNS, in accordance with the IHC data.

Caspase 1 was increased in mild-moderate COPD compared to both CNS and CS subjects, at variance with the IHC localized quantifications performed in the bronchioles and alveolar septa. SERPINs B3, B5, B11, B13, tPA and cathepsin L did not change significantly among groups.

### 2.5. Principal Correlations Between Clinical Parameters, SERPINs and Proteases in Lung Parenchyma

**Figure 4:**
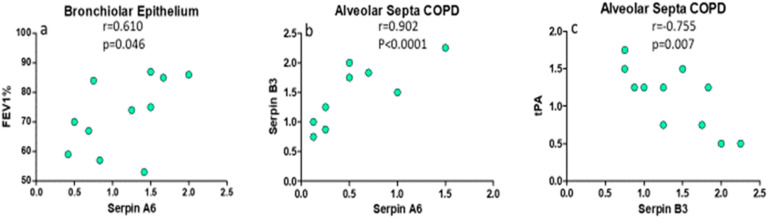
Regression analysis in COPD patients alone: (**a**) in these patients, there was a significant positive correlation between SERPINA6 in bronchiolar epithelium and the predicted post-bronchodilator FEV1%; (**b**) in alveolar septa, SERPINA6 was positively correlated to SERPINB3; and (**c**) SERPINB3 was inversely correlated to tPA. Correlation coefficients were calculated by using the Spearman rank method.

In COPD patients, there was a significant positive correlation between SERPINA6 in bronchiolar epithelium and the predicted post-bronchodilator FEV1% (R = 0.610, *p* = 0.046, Figure 4); in alveolar septa, SERPINA6 was positively correlated to SERPINB3 (R = 0.902, *p* < 0.0001, Figure 4), and SERPINB3 was inversely correlated to tPA values (R = −0.755, *p* = 0.0072, Figure 4).

## 3. Discussion

To our knowledge, this is the first study that has comprehensively analyzed the gene and protein expression of all members of the SERPIN family in different lung compartments in COPD, in comparison to two control groups including CS and CNS. The SERPIN family is a large group of antiproteases involved in several biological functions and is composed of 37 members. To avoid the analysis of SERPINs not expressed in lung tissue, we first analyzed all members of the SERPIN family through a transcriptomic methodological approach to evaluate the mRNA levels for each SERPIN amongst all groups. We found significant different expressions in terms of mRNA levels for 15 members of the SERPIN family. These molecules were then analyzed through both IHC and ELISA.

Specifically, one of the relevant results is the decreased expression of SERPINA6 in CS (which also tends to decrease in the COPD group) compared to that in CNS. This molecule, also termed corticosteroid-binding globulin (CBG), is usually an extracellular protein that can bind and transport glucocorticoids [28]. Impaired expression of SERPINA6 is correlated with cardiovascular and metabolic diseases [28,29]. A decreased expression of SERPINA6 in COPD patients was correlated with impaired expression of SERPINA1 [4,30]. Our result confirms these data, showing that the expression of this molecule is decreased in COPD, differing from other inflammatory diseases such as asthma [31]. Our result was found in several lung compartments, as was also confirmed through ELISA analysis. In addition, we also found a positive correlation between CBG expression and lung function (FEV1%), reinforcing the notion of a CBG decrement in relation to the severity of the COPD. These data may relate to clinical reports on the decreased effectiveness of ICS, particularly in COPD patients who continue to smoke.

Regarding the clade B of SERPINs, we found impaired expression of several isoforms including B13, B5, B3 and B11.

Increased SERPINB13 expression was found in COPD/CS patients compared to the CNS group in several lung compartments, including alveolar septa and lung vessels. It is known that SERPINB13 has a role in cell proliferation and differentiation. Moreover, an association between SERPINB13 gene expression and squamous cell carcinoma was found [32,33]. Our data may represent another link between the presence of COPD and lung cancer development [34].

This hypothesis is also reinforced by another result found in our analysis showing increased expression of SERPINB5, specifically in the CS group. Although the role of this molecule is largely unknown, SERPINB5 seems to act as a tumor suppressor through the inhibition of cancer cell migration, invasion capability, and angiogenesis, suggesting a compensatory mechanism in CS and COPD patients in the relationship between COPD and lung cancer [33,34,35,36].

We found no significant differences in SERPINB3 expression amongst all groups in the major part of the analyzed lung compartments; we found it was decreased in the alveolar septa of COPD/CS patients compared to the CNS group. Recent data have shown that SERPINB3 has a protective role in mediating tissue repair, especially during hypoxic conditions, through its proliferative action [37], suggesting a role in the development of pulmonary emphysema through the decreased repair capability in lung tissue of these patients.

We found increased cathepsin L both at the protein and mRNA levels. The IHC analysis showed increased levels in alveolar septa and alveolar macrophages in COPD/CS patients compared to the CNS group. Cathepsins, in particular, cathepsin L expression, is regulated by the SERPINB group, and particularly by SERPINB3; thus, the decreased expression of this molecule in alveolar septa could be related to the increased expression of cathepsin L, which is able to modulate the lysosome-mediated intracellular protein degradation. Cathepsin L has a key role in phagocytosis and intracellular protein degradation; this can be related to both pulmonary emphysema development and the impaired alveolar macrophages functions reported in COPD [36].

Another important finding is the increased expression of SERPINB11 in the alveolar septa and lung vessels in COPD patients compared to the CS group. Although the role of SERPINB11 is still now largely unclear, there is some evidence that this molecule is increased in several types of cancer, correlating with poor patient prognosis and therapy response [37].

We also found an increased expression of PLAUR in the alveolar macrophages of both COPD and CS groups, which is in line with other data in the literature [16]. Although PLAUR has an important fibrinolytic role similar to that of tPA, this molecule plays a key role in inflammation, cell adhesion, and chemotaxis; pre-clinical data demonstrated that decreased levels of PLAUR are correlated to decreased lung inflammation [38].

Finally, both tPA and PLAUR, which were shown to have increased in several lung compartments in this study, are modulated and inhibited by SERPINB2. The lack of an increase in SERPINB2, in contrast to the tPA and PLAUR activity, may have a role in increased inflammation in COPD patients.

We did not find any significant variations in SERPINC1 and SERPIND1 in the different lung compartments analyzed by IHC, which is in contrast to the reduced SERPINC1 activity [11] previously reported in exacerbated COPD. This may be due to the different disease states of the patients studied.

We found a discrepancy between some IHC and ELISA data; in fact, not all IHC data were confirmed by ELISA. We speculate that this may be due to the different methodological approaches; ELISA tests represent the total amount of lung proteins extracts while IHC results are obtained specifically in different lung compartments.

Finally, our transcriptomic analysis showed a clear prevalence of proteases expression (Figure 1) in the lungs of all patients together with an imbalance in SERPINs, mainly observed at the post-transcriptional level. This is in line with our experience, as there are frequently discrepancies between transcriptomic and protein data [39].

In different lung compartments of COPD patients, we reported decreased levels of SERPINB3, unchanged levels of SERPINB2 concomitant to increased levels of some related proteases such as cathepsin L, PLAUR and tPA. This study highlights the most important SERPINs and related proteases involved in the pathogenesis of stable COPD of increasing severity.

## 4. Materials and Methods

### 4.1. Subjects

All COPD and healthy control subjects were recruited from the Respiratory Medicine Unit of the San Luigi Gonzaga University Hospital (Orbassano-Turin, Italy). Archival material was used in the present study [40]. Thirty-nine subjects undergoing lung resection for a solitary peripheral neoplasm were recruited for the immunohistochemical study of peripheral lung tissue and the transcriptomic analysis of lung tissue. The characteristics of these subjects are reported in Table 1.

All COPD patients were stable and none of the COPD subjects were treated with theophylline, antibiotics, antioxidants, mucolytics, and/or glucocorticoids in the month prior to lung resection surgery. The study conformed to the Declaration of Helsinki; the study was approved by the Institutional Review Boards of the Istituti Clinici Scientifici Maugeri (protocol p112) and by the Ethical Committee of the San Luigi Gonzaga University Hospital (protocol n. 9544/2019). More details on the characteristics of the subjects are reported in the Supplementary Materials.

### 4.2. Lung Function Tests and Volumes

Pulmonary function tests were performed in all subjects according to current guidelines. The severity of the airflow obstruction in COPD patients was staged using GOLD criteria [https://goldcopd.org/wp-content/uploads/2021/12/GOLD-REPORT-2022-v1.1-22Nov2021_WMV.pdf (accessed on 20 December 2024)] on the basis of pulmonary function tests.

### 4.3. Collection and Processing of the Peripheral Lung Tissue

Thirty-nine subjects undergoing lung resection surgery for a solitary peripheral neoplasm were recruited. Twelve were nonsmokers with normal lung function, 14 subjects were smokers with normal lung function, and 13 were smokers with COPD (Table 1). All former smokers had stopped smoking for more than one year. All subjects did not undergo preoperative chemotherapy and/or radiotherapy and had not been treated with bronchodilators, theophylline, antibiotics, antioxidants, and/or glucocorticoids in the month prior to surgery. Lung tissue processing was performed as previously described [40,41] Two to four randomly selected tissue blocks were taken from the lung obtained at surgery, avoiding areas grossly invaded by tumors. Samples were fixed in 4% formaldehyde in phosphate-buffered saline at a pH of 7.2 or frozen in liquid nitrogen. Fixed specimens, after dehydration, were embedded in paraffin wax. Serial sections that were 6 µm-thick from frozen specimens were first cut and stained with hematoxylin–eosin (H and E) in order to visualize the morphology and to exclude the presence of microscopically evident tumor infiltration. Frozen tissue specimens were used in this study. Specimens were then cut for immunohistochemical analysis and were placed on charged slides, as previously reported [40,41].

### 4.4. Immunohistochemistry in Human Peripheral Lung Tissue

Immunostaining of frozen peripheral lung tissue was performed as previously described [40,41]. In the present study, frozen sections were used for immunohistochemical analysis.

Endogenous peroxidase activity was blocked by incubating slides in 3% hydrogen peroxide (H_2_O_2_) in phosphate-buffered saline (PBS) followed by washing in PBS. Cell membranes were permeabilized by adding 0.1% saponin to the PBS. Non-specific labeling was blocked by coating the slides with blocking serum (5% normal animal serum) for 20 min at room temperature. After washing in PBS, the sections were incubated with primary antibodies (Table S1). Control slides were included in each staining run using human tonsils or nasal polyps as a positive control for all the immunostaining performed. Slides were then incubated with chromogen-fast diaminobenzidine (DAB) as the chromogenic substance, after which they were counterstained in hematoxylin and mounted on a permanent mounting medium.

### 4.5. Scoring System for Immunohistochemistry in the Peripheral Lung Tissue

All disposable bronchioles, alveolar macrophages, alveolar septa and vessels observed in each lung section specimen were analyzed for each immunostained section. The immunopositivity was scored: 0, absence of immunostaining, 1, 33% of immunostained cells, 2, 66% of immunostained cells, and 3 (almost all positive cells). The intensity of the immunopositivity was considered by adding a 0.5 score point to the established score applied on the basis of the number of positive cells in the bronchiolar epithelium, bronchiolar lamina propria, alveolar macrophages, alveolar septa, and lung vessels [40,41]. The minimum internal diameter was measured in one adjacent section stained with H and E to identify the bronchial level studied and the total number of bronchioles analyzed for each subject included in the study.

### 4.6. ELISA Tests in the Peripheral Lung Tissue Homogenates

SERPINA6 (MyBioSource, San Diego, CA, USA Cat. N. MBS700017, lower detection limit, 20 pmol/mL), SERPINB3 (Antibodies-online, Aachen, Germany Cat. N. ABIN6574275, lower detection limit, 33 pg/mL), SERPINB5 (MyBioSource, San Diego, CA, USA, Cat. N. MBS9426741, lower detection limit, 0.78 ng/mL), SERPINB11 (MyBioSource, San Diego, CA, USA Cat. N. MBS9313345, lower detection limit, 0.1 ng/mL), SERPINB13 (MyBioSource, San Diego, CA, USA Cat. N. MBS1607013, lower detection limit, 9.33 ng/L), TPA (MyBioSource, San Diego, CA, USA Cat. N. MBS161723, lower detection limit, 0.05 ng/mL), caspase1 (Antibodies-online, Aachen, Germany Cat. N. ABIN 6574284, lower detection limit, 0.113 ng/mL), cathepsin L (Antibodies-online, Aachen, Germany Cat. N. ABIN 6954461, lower detection limit, 0.122 ng/mL) protein quantification was performed in the lung tissue homogenates obtained from the frozen tissue specimens that were also used for the immunohistochemical analysis. All ELISA kits were used according to the manufacturer’s instructions.

### 4.7. RNA Extraction and Sequencing from Lung Specimens

Frozen lung parenchymal tissue used for the immunohistochemical analysis; bronchial rings from the same patients were also used for RNA extraction, sequencing and gene expression data analysis.

RNA extraction was performed with the RNAeasy micro kit (Qiagen, Hilden, Germany) following the manufacturer’s instructions. A DNA removal step was applied using 500 units of RNase–free DNase (Qiagen) at room temperature for 15 min. Total RNA was resuspended in RNase-free water (Thermo Fisher, Carlsbad, CA, USA) and the RNA/DNA concentrations in each sample were quantified using the Qubit RNA and DNA High-Sensitivity Assay Kit (Thermo Fisher). RNA qualities were assessed with an Agilent Bioanalyzer 2100 equipped with an RNA nano 6000 kit (Agilent, Santa Clara, CA, USA). Due to the low RIN values obtained for lung parenchyma samples, RNA-sequencing libraries for these samples were prepared following a 3′-end sequencing procedure using the QuantSeq 3′ mRNA-Seq Library Prep Kit FWD for Illumina (Lexogen, Vienna, Austria). Consequently, lung parenchyma libraries were sequenced using an Illumina NextSeq500 (Cribi, UniPD, Padova, Italy) with a 75-single-end read layout. In contrast, no quality issues were encountered for the bronchial ring samples; therefore, standard Illumina library preparation methods were performed. Bronchial ring libraries were then sequenced with a 150-paired-end read layout (Cribi).

### 4.8. Data Analysis of RNA-Seq Data

The raw Illumina reads were trimmed for quality using fastp [42], setting a minimal Phred quality of 25 and removing the sequencing adaptors. Raw Illumina datasets were submitted to the NCBI Short Read Archive (SRA) under the project ID PRJNA1041288. FASTQ files were imported using CLC Genomic Workbench, v. 21 (Qiagen, Hilden, Denmark) and analyzed as follows. To identify differentially expressed genes (DEGs), the trimmed reads were mapped on the human reference genome (hg19, Ensembl, v.99) applying the following parameters: Mismatch cost = 2; Insertion cost = 3; Deletion cost = 3; Length fraction = 0.8; Similarity fraction = 0.8; and strand-specific mapping. Expression values were counted as transcripts per millions (TPMs). A Baggerley test with a false discovery rate (FDR) *p*-value correction was applied to identify differentially expressed genes (DEGs), setting a cutoff of 2 logarithmic fold changes (FC) and 0.01 of the *p*-value. Limited to the gene of interest, the expression levels were extracted from the overall dataset and further discussed.

### 4.9. Statistical Analysis Applied to Functional and Morphological Data

Group data were expressed as mean (standard deviation) for functional data or median (range) or interquartile range (IQR) for morphologic data. Differences between groups were analyzed using analysis of variance (ANOVA) for functional data. ANOVA was followed by an unpaired t-test for comparison between groups. The Kruskal–Wallis test was applied to the morphologic data, followed by the Mann–Whitney U-test for comparison between groups. Correlation coefficients were calculated using the Spearman rank method. Probability values of *p* < 0.05 were considered significant. Data analysis was performed using the Stat View SE Graphics program (Abacus Concepts Inc., Berkeley, CA, USA).

## Figures and Tables

**Table 1:** Clinical characteristics of subjects for immunohistochemistry studies on peripheral lung tissue.

Grups	Control Nonsmokers	Control Smokers	Patients with COPD
N°	12	14	13
Age (y)	66 ± 4.6	67 ± 1.6	70 ± 1.7
M/F	5/7	9/5	11/2
Ex/Current Smokers	---	9/5	10/3
Pack Years	---	37 ± 4.8	54 ± 11
FEV1 (% pred) pre-β2	113 ± 4.2	97 ± 2.9	73 ± 4.7 #
FEV1 (% pred) post-β2	ND	ND	82 ± 5.2
FEV1/FVC (%) pre-β2	81 ± 1.4	73 ± 1.0	58 ± 2.4 #
TLC (%)	105.2 ± 3.5	102.7 ± 2.1	114.6 ± 3.9 *
RV (%)	109.0 ± 6.5	114.6 ± 3.7	150.7 ± 8.7 #
DLCO (%)	ND	90.4 ± 3.6	80.6 ± 5.5

Data are expressed as mean ± SE. Patients with chronic obstructive pulmonary disease (COPD) were classified according to GOLD 2022 (goldcopd.org) grades of severity using only the severity of airflow obstruction. For COPD patients, FEV1/FVC (%) are post-bronchodilator values. Abbreviations: M, male; F, female, FEV1: forced expiratory volume in one second; FVC, forced vital capacity; ND, not determined; Statistical analysis: ANOVA test #, *p* < 0.0001, significantly different from control smokers with normal lung function and control never smokers; *, *p* < 0.010 significantly different from control smokers with normal lung function. TLC%, RV%, DLCO% all values at baseline.

**Table 2:** Immunohistochemical quantification of SERPIN-signaling proteins in the lung parenchyma.

Localization	Control Nonsmokers	Control Smokers	COPD Patients	Kruskal Wallis *p*-Value
Bronchiolar Epithelium (score 0–3)				
SERPINA3	1.0 (0.25–1.5)	0.75 (0.5–1.5)	0.75 (0.25–1.5)	0.673
SERINA6	1.58 (0.75–2.0)	0.81 (0.25–1.36) *	1.25 (0.41–2.0)	**0.025**
SERPINB2 (PAI-2)	0.25 (0.0–0.625)	0.125 (0.0–0.625)	0.25 (0.0–0.5)	0.968
SERPINB3	2.56 (2.0–3.0)	2.4 (1.0–2.5)	2.56 (2.0–2.95)	0.116
SERPINB5	2.45 (1.25–3.0)	3.0 (1.75–3.0) *	2.75 (1.75–3.0) &	**0.016**
SERPINB11	2.75 (2.33–3.0)	2.5 (1.37–2.87)	2.87 (2.33–3.0)	0.163
SERPINB13	0.44 (0.0–0.62)	0.77 (0.0–1.5)	0.67 (0.0–2.0)	0.130
SERPINC1	3.0 (1.8–3.0)	2.83 (1.5–3.0)	3.0 (2.5–3.0)	0.244
SERPIND1	0.0 (0.0–0.0)	0.0 (0.0–0.0)	0.0 (0.0–0.1)	0.621
SPINK1	2.96 (1.5–3.0)	2.75 (1.83–3.0)	2.95 (2.5–3.0)	0.205
PLAUR (UPAR)	2.75 (1.25–3.0)	2.95 (1.75–3.0)	2.75 (1.5–3.0)	0.199
tPA	1.75 (0.75–2.87)	2.75 (0.75–3.0) *	2.75 (0.5–3.0) *	**0.050**
Cathepsin K	0.55 (0.0–1.75)	0.75 (0.0–1.12)	0.5 (0.15–1.16)	0.772
Cathepsin L	2.25 (0.75–3.0)	2.75 (1.5–3.0)	2.87 (1.75–3.0)	0.158
Caspase1	2.92 (2.75–3.0)	2.5 (1.5–3.0)	2.85 (1.5–3.0)	0.122
Bronchiolar Lamina Propria (score 0–3)				
SERPINA3	1.0 (0.25–1.5)	0.75 (0.5–1.5)	0.75 (0.25–1.5)	0.789
SERPINA6	1.0 (0.5–1.5)	0.5 (0.0–1.0) *	0.5 (0.25–1.5) *	**0.029**
SERPINB2 (PAI-2)	0.0 (0.0–0.5)	0.5 (0.0–1.0)	0.5 (0.0–0.5)	0.590
SERPINB3	1.0 (0.75–2.5)	1.0 (0.5–1.0)	1.0 (1.0–2.5)	0.128
SERPINB5	2.41 (1.25–3.0)	2.87 (1.75–3.0) *	2.75 (1.75–3.0)	**0.065**
SERPINB11	2.5 (2.0–2.92)	2.25 (1.25–2.75)	2.5 (2.0–2.5)	0.252
SERPINB13	0.12 (0.0–0.5)	0.5 (0.0–1.0) *	0.5 (0.0–2.0) *	**0.027**
SERPINC1	2.5 (1.5–2.87)	2.5 (1.0–3.0)	2.75 (2.0–3.0)	0.099
SERPIND1	0.0 (0.0–0.12)	0.0 (0.0–0.0)	0.0 (0.0–0.05)	0.582
SPINK1	2.5 (1.5–2.5)	2.5 (1.5–2.75)	2.5 (2.0–2.5)	0.580
PLAUR (UPAR)	2.5 (1.25–2.75)	2.5 (1.75–3.0)	2.5 (1.5–2.75)	0.700
tPA	1.0 (0.5–2.5)	2.0 (0.5–2.5)	2.25 (0.5–2.5)	0.160
Cathepsin K	0.25 (0.0–0.5)	0.25 (0.0–0.5)	0.25 (0.0–0.5)	0.917
Cathepsin L	2.0 (0.5–2.5)	2.5 (1.5–3.0)	2.5 (1.5–2.5)	**0.201**
Caspase1	2.75 (2.5–3.0)	2.0 (1.0–3.0) *	2.5 (1.5–3.0)	0.021
Alveolar Macrophages (score 0–3)				
SERPINA3	0.75 (0.25–1.36)	1.0 (0.5–1.5)	0.75 (0.5–1.75)	0.319
SERPINA6	1.75 (0.5–2.0)	1.25 (0.5–2.0)	1.0 (0.41–2.0)	0.266
SERPINB2 (PAI-2)	0.5 (0.0–2.25)	0.89 (0.1–2.0)	0.5 (0.25–2.0)	0.322
SERPINB3	1.5 (0.75–2.5)	1.25 (0.75–2.25)	1.0 (0.5–2.25)	0.482
SERPINB5	2.0 (1.5–2.5)	2.5 (1.75–3.0) *	2.25 (1.83–3.0)	**0.017**
SERPINB11	2.25 (1.5–2.87)	2.0 (1.5–2.5)	2.25 (1.25–2.75)	0.093
SERPINB13	1.0 (0.12–2.0)	2.0 (0.17–2.5) *	2.0 (0.17–2.5) *	**0.012**
SERPINC1	3.0 (2.5–3.0)	2.75 (2.0–3.0)	2.87 (2.25–3.0)	0.140
SERPIND1	0.04 (0.0–0.57)	0.06 (0.0–1.0)	0.07 (0.0–0.25)	0.708
SPINK1	2.5 (2.0–2.92)	2.5 (2.0–3.0)	2.5 (1.0–3.0)	0.778
PLAUR (UPAR)	2.5 (2.0–3.0)	3.0 (2.0–3.0) *	2.75 (1.83–3.0) &	**0.026**
tPA	1.75 (0.75–2.5)	2.0 (1.0–2.75)	2.0 (0.75–2.75)	0.215
Cathepsin K	1.5 (0.25–2.25)	1.75 (0.5–2.5)	1.37 (0.2–2.25)	0.617
Cathepsin L	2.25 (1.25–2.75)	2.75 (1.75–3.0) *	2.75 (1.5–3.0) *	**0.063**
Caspase1	2.75 (2.25–3.0)	2.5 (1.5–3.0)	2.5 (2.25–3.0)	0.111
Alveolar Septa (score 0–3)				
SERPINA3	1.0 (0.5–1.7)	0.83 (0.5–1.33)	0.79 (0.5–125)	0.385
SERPINA6	0.75 (0.0–1.5)	0.375 (0.0–1.5)	0.25 (0.125–1.5)	**0.080**
SERPINB2 (PAI-2)	1.0 (0.125–1.5)	1.0 (0.08–1.5)	1.25 (0.25–2.0)	0.604
SERPINB3	1.5 (1.0–2.5)	1.25 (0.75–1.5) *	1.25 (0.75–2.25)	**0.058**
SERPINB5	1.5 (1.0–2.5)	2.25 (1.5–3.0) *	2.0 (1.5–2.75) *	**0.029**
SERPINB11	1.87 (1.5–2.5)	1.5 (1.0–2.0) *	1.9 (1.25–2.5) &	**0.015**
SERPINB13	0.25 (0.08–0.83)	1.0 (0.16–1.5) *	0.75 (0.0–2.0) *	**0.022**
SERPINC1	1.91 (1.5–2.5)	1.5 (1.0–2.5)	1.75 (1.42–2.75)	0.533
SERPIND1	0.0 (0.0–0.29)	0.0 (0.0–0.25)	0.0 (0.0–0.25)	0.576
SPINK1	1.5 (1.0–2.0)	1.5 (1.0–2.0)	1.75 (1.0–2.5)	0.466
PLAUR (UPAR)	2.0 (1.0–2.0)	1.5 (1.0–2.0)	1.5 (1.0–2.5)	0.628
tPA	0.5 (0.25–1.5)	1.0 (0.25–2.0)	1.25 (0.5–1.75)	0.112
Cathepsin K	0.5 (0.0–1.0)	0.5 (0.25–1.0)	0.37 (0.25–0.75)	0.155
Cathepsin L	1.4 (0.5–2.25)	2.0 (1.0–2.5)	2.0 (1.25–2.5) *	**0.043**
Caspase1	2.5 (2.0–3.0)	2.0 (1.0–3.0) *	2.5 (2.0–2.75)	**0.046**
Lung Vessels (score 0–3)				
SERPINA3	1.12 (0.5–1.5)	1.0 (0.5–1.5)	1.0 (0.5–1.75)	0.256
SERPINA6 e	0.5 (0.0–1.0)	0.0 (0.0–1.0) *	0.375 (0.0–1.0)	**0.063**
SERPINB2 e (PAI-2)	0.5 (0.0–1.0)	0.5 (0.0–1.5)	0.5 (0.0–1.0)	0.801
SERPINB3 e	0.75 (0.5–1.5)	0.75 (0.5–1.0)	0.75 (0.5–1.0)	0.973
SERPINB5 e	1.5 (1.0–2.0)	1.5 (1.0–2.5)	1.75 (1.25–2.5)	0.156
SERPINB11 e	1.5 (1.5–2.5)	1.5 (0.75–2.0) *	2.0 (1.0–2.5) &	**0.045**
SERPINB13 e	0.5 (0.0–0.75)	1.0 (0.0–1.25) *	0.75 (0.0–1.5) *	**0.013**
SERPINC1	2.0 (2.0–2.5)	2.0 (1.0–2.5)	2.0 (1.0–2.5)	0.139
SERPIND1	0.0 (0.0–0.0)	0.0 (0.0–0.0)	0.0 (0.0–0.0)	NV
SPINK1 e	1.5 (1.0–2.0)	1.5 (1.0–1.5)	1.5 (1.0–2.0)	0.611
PLAUR (UPAR)	1.62 (1.0–2.0)	1.5 (1.25–2.0)	1.5 (1.0–2.5)	0.676
tPA	0.87 (0.0–1.5)	1.0 (0.25–1.75) *	1.25 (0.5–2.0) *	**0.055**
Cathepsin K	0.5 (0.0–1.5)	0.75 (0.0–1.0)	0.5 (0.0–1.0)	0.374
Cathepsin L e	1.12 (0.25–2.0)	1.25 (1.0–2.5)	1.5 (0.5–2.0)	0.399
Caspase1 e	2.25 (1.25–2.5)	1.5 (1.0–2.5) *	1.75 (1.5–2.5) *	**0.010**

Abbreviations: COPD, chronic obstructive pulmonary disease. Data are expressed as median (range). Statistics: the Kruskal–Wallis test was used for multiple comparisons followed by the Mann–Whitney U test for comparison between groups: *, *p* < 0.05, significantly different from control nonsmokers; &, *p* < 0.05, significantly different from control smokers with normal lung function. Lung vessels-e, endothelial immunopositivity in the lung vessels. The exact “*p*” values for comparison between groups are given in the results section and/or figures.

## Data Availability

The data from this study are available upon reasonable request.

## Supplementary Materials

The following supporting information can be downloaded at: https://www.mdpi.com/article/10.3390/ijms26072832/s1.
