# Supplementary material for: Impaired SERPIN–Protease Balance in the Peripheral Lungs of Stable COPD Patients
Source: Int J Mol Sci. 2025 Mar 21;26(7):2832. doi: 10.3390/ijms26072832 (PMC11988695; doi:10.3390/ijms26072832)
Supplement: Supplementary file 1 [file ijms-26-02832-s001.zip › ijms-3458604-supplementary.pdf]

Supplementary Materials

Impaired serpin-protease balance in the peripheral lung of stable COPD patients

Antonino Di Stefano[1]*, Francesco Nucera[2]*, Umberto Rosani[3], Paola Brun[4], Isabella Gnemmi[1], Mauro Maniscalco[5], Silvestro Ennio D'Anna[5], Andrea Leonardi[6], Vitina Carriero[7], Francesca Bertolini[7], Josè Freni[2], Antonio Ieni[8], Sebastiano Gangemi[9], Paolo Ruggeri[2#], Fabio Luigi Massimo Ricciardolo[7#]

[1] Divisione di Pneumologia e Laboratorio di Citoimmunopatologia dell'Apparato Cardio Respiratorio, Istituti Clinici Scientifici Maugeri, IRCCS, Respiratory Rehabilitation Unit of Gattico-Veruno, 28013, Novara (NO), Italy
[2] Pneumologia, Dipartimento di Scienze Biomediche, Odontoiatriche e delle Immagini Morfologiche e Funzionali (BIOMORF), Università degli Studi di Messina, Piazza Pugliatti 1, 98122 Messina, Italy;
[3] Department of Biology, University of Padova, via U. Bassi 58/b, 35121 Padova; Italy
[4] Department of Molecular Medicine, Histology Unit, University of Padova, 35121, Padova, Italy;
[5] Divisione di Pneumologia, Istituti Clinici Scientifici Maugeri, IRCCS, Telese, 82037, Benevento (BN), Italy;
[6] Ophthalmology Unit, Department of Neuroscience, University of Padova, Padova, Italy.
[7] Department of Clinical and Biological Sciences, University of Turin, Severe Asthma, Rare Lung Disease and Respiratory Pathophysiology Unit, San Luigi Gonzaga University Hospital, Orbassano, 10043, Turin, Italy;
[8] Department of Human Pathology in Adult and Developmental Age 'Gaetano Barresi', Section of Pathology, University of Messina, 98122 Messina, Italy.
[9] Operative Unit of Allergy and Clinical Immunology Department of Clinical and Experimental Medicine, University of Messina, 98125 Messina, Italy

*These authors share the first author position
#These authors share the last author position

Short Title: Serpins signalling in COPD

Corresponding Author:

Antonino Di Stefano, PhD
E-mail: antonino.distefano@icsmaugeri.it

Ricciardolo Fabio Luigi Massimo, MD PhD FERS
E-mail: fabioluigimassimo.ricciardolo@unito.it

Table S1. Primary antibodies and immunohistochemical conditions used for identification of Serpins signaling proteins in peripheral lung

Target	Supplier	Cat.#[a]	Source	Dilution	Positive control
Serpin A3	ThermoFisher	PA5-120834	Rabbit	1:800	Nasal polyp, human tonsil
Serpin A6	LSBio	LS-B10686	Rabbit	1:50	Nasal polyp, Human tonsil
Serpin B2 (PAI-2)	ThermoFisher	PA5-27857	Rabbit	1:200	Nasal polyp, human tonsil
Serpin B3	ThermoFisher	PA5-30164	Rabbit	1:200	Nasal polyp, human tonsil
Serpin B5	LSBio	LS-C164042	Rabbit	1:50	Nasal polyp, human tonsil
Serpin B11	LSBio	LS-C770539	Rabbit	1:50	Nasal polyp, human tonsil
Serpin B13	BiossAntibodies	BS-8324R	Rabbit	1:100	Nasal polyp, human tonsil
Serpin C1	ThermoFisher	PA5-13674	Rabbit	1:100	Nasal polyp, human tonsil
Serpin D1	Santa Cruz	SC-69784	Mouse	1:500	Nasal polyp, Human tonsil
SPINK1	BiossAntibodies	BS-2916R	Rabbit	1:50	Nasal polyp, Human tonsil
PLAUR (UPAR)	BiossAntibodies	BS-1927R	Rabbit	1:100	Nasal polyp, Human tonsil
tPA	ThermoFisher	PA5-95629	Rabbit	1:250	Nasal polyp, Human tonsil
Cathepsin K	ThermoFisher	PA5-102483	Rabbit	1:100	Nasal polyp, Human tonsil
Cathepsin L	ThermoFisher	PA5-119012	Rabbit	1:100	Nasal polyp, Human tonsil
Caspase 1	ThermoFisher	MA5-16215	Mouse	1:100	Nasal polyp, Human tonsil

[a]Cat#, catalogue number

Table S2		CS vs CNS		CS vs COPD		COPD vs CNS		CNS_mean	COPD_mean	CS_mean
**Gene ID**	**Database object name**	**Log FC**	**P-value**	**Log FC**	**P-value**	**Log FC**	**P-value**			
CASP1	Caspase-1	0,09	0,74	0,13	0,61	-0,04	0,86	42,58	41,22	46,58
CASP10	Caspase-10	0,12	0,63	0,01	0,97	0,11	0,63	18,55	20,06	20,96
CASP4	Caspase-4	-0,02	0,94	-0,22	0,35	0,2	0,36	75,4	88,35	78,78
CASP8	Caspase-8	0,27	0,33	0,18	0,5	0,09	0,73	25,31	26,88	31,21
CEBPA	CCAAT/enhancer-binding protein alpha	-0,23	0,57	0,3	0,43	-0,53	0,15	34,23	23,41	29,13
CELA1	Chymotrypsin-like elastase family member 1	1,29	0,37	-1,26	0,29	2,55	0,04	0,03	0,25	0,11
CELA2A	Chymotrypsin-like elastase family member 2A	1,08	0,45	-0,54	0,67	1,62	0,21	0,06	0,21	0,14
CELA2B	Chymotrypsin-like elastase family member 2B	-0,57	0,63	-1,7	0,14	1,13	0,28	0,44	0,87	0,27
CMA1	Chymase	-1,14	0,3	-1,13	0,3	-6,37E-03	0,99	0,55	0,49	0,23
CTRC	Chymotrypsin-C	-3,34	0,03	-0,96	0,56	-2,38	0,04	0,41	0,06	0,03
CTRL	Chymotrypsin-like protease CTRL-1	0,56	0,49	0,48	0,54	0,08	0,91	1,08	1,23	1,7
CTSG	Cathepsin G	-0,26	0,68	-1	0,1	0,74	0,2	3,3	5,61	2,94
CTSK	Cathepsin K	-0,47	0,31	-0,84	0,06	0,37	0,39	22,89	28,86	17,14
CTSL	Cathepsin L1	0,07	0,83	0,26	0,4	-0,19	0,52	110,18	96,18	118,21
CTSS	Cathepsin S	0,14	0,64	-0,15	0,61	0,3	0,3	277,8	341,1	308,45
ELANE	Neutrophil elastase	-0,84	0,37	-0,64	0,48	-0,19	0,82	0,63	0,53	0,37
F2R	Proteinase-activated receptor 1	-0,36	0,26	-0,52	0,09	0,17	0,57	44,98	49,3	35,67
F2RL1	Proteinase-activated receptor 2	-0,05	0,94	0,1	0,88	-0,14	0,8	1,68	1,48	1,65
GZMB	Granzyme B	0,02	0,97	-0,49	0,25	0,51	0,21	19,56	27,8	20,26
KLK2	Kallikrein-2	2,57	0,1	1,95	0,17	0,62	0,71	0,02	0,04	0,26
KLK3	Prostate-specific antigen	-0,04	0,99	-2,55	0,23	2,5	0,16	0	0,05	0
NPM2	Nucleoplasmin-2	1,7	0,03	-0,08	0,92	1,78	0,01	1,1	4,14	3,95
PLAT	Tissue-type plasminogen activator	0,01	0,97	-0,1	0,77	0,11	0,72	40,65	44,96	42,86
PLAU	Urokinase-type plasminogen activator	-0,38	0,28	0,23	0,5	-0,61	0,06	13,63	9,09	10,69
PLAUR	Urokinase plasminogen activator surface receptor	-0,2	0,62	0,2	0,61	-0,39	0,28	108,44	85,95	100,61
SERPINA1	Alpha-1-antitrypsin	0,51	0,14	0,3	0,36	0,21	0,5	126,05	145,51	185,77
SERPINA10	Protein Z-dependent protease inhibitor	NaN	NaN	NaN	NaN	NaN	NaN	0	0	0
SERPINA11	Serpin A11	2,19	0,22	2,18	0,23	0,01	1	0	0	0,05

SERPINA3	Alpha-1-antichymotrypsin	**-2,32**	**6,02E-04**	-0,29	0,66	**-2,03**	**1,07E-03**	221,56	60,56	51,53
SERPINA5	Plasma serine protease inhibitor	0,9	0,37	0,53	0,59	0,37	0,69	0,5	0,68	0,99
SERPINA6	Corticosteroid-binding globulin	1,38	0,27	1,37	0,27	7,14E-03	1	0,06	0,05	0,15
SERPINA9	Serpin A9	-0,02	0,99	-0,39	0,76	0,37	0,76	0,16	0,19	0,14
SERPINB1	Leukocyte elastase inhibitor	-0,18	0,52	-0,09	0,74	-0,09	0,73	177,4	171,37	164,32
SERPINB10	Serpin B10	3,06E-03	1	-0,36	0,77	0,37	0,75	0,11	0,13	0,1
SERPINB11	Serpin B11	**5,28**	**8,22E-03**	0,25	0,84	5,03	9,42E-03	0	0,49	0,61
SERPINB12	Serpin B12	NaN	NaN	NaN	NaN	NaN	NaN	0	0	0
SERPINB13	Serpin B13	4	5,28E-03	2,43	0,06	1,57	0,26	0,08	0,26	1,51
SERPINB2	Plasminogen activator inhibitor 2	1,87	0,13	2,4	0,05	-0,54	0,65	0,5	0,38	2,08
SERPINB3	Serpin B3	1,25	0,27	**2,65**	**0,02**	-1,4	0,19	1,95	0,8	5,32
SERPINB4	Serpin B4	2,17	0,28	-0,32	0,82	2,49	0,19	0	0,07	0,05
SERPINB5	Serpin B5	-0,42	0,74	**3,4**	**0,01**	**-3,82**	**2,67E-03**	2,29	0,17	1,98
SERPINB6	Serpin B6	0,09	0,69	0,11	0,6	-0,02	0,9	76,07	75,2	84,48
SERPINB7	Serpin B7	2,99	0,12	0,82	0,53	2,17	0,25	0	0,05	0,1
SERPINB8	Serpin B8	0,02	0,96	-0,06	0,9	0,08	0,85	5,06	5,68	5,36
SERPINB9	Serpin B9	-0,22	0,48	-0,37	0,23	0,15	0,61	25	28,48	22,89
SERPINC1	Antithrombin-III	-0,02	0,99	-0,06	0,96	0,04	0,98	0,17	0,17	0,16
SERPIND1	Heparin cofactor 2	**2,19**	**8,56E-03**	-0,9	0,25	**3,09**	**5,31E-05**	0,71	5,88	3,28
SERPINE1	Plasminogen activator inhibitor 1	-0,12	0,84	-0,57	0,3	0,45	0,39	160,32	234,96	163,87
SERPINE2	Glia-derived nexin	-0,37	0,37	-0,55	0,16	0,18	0,62	13,12	15,08	10,6
SERPINF1	Pigment epithelium-derived factor	-0,35	0,36	-0,58	0,12	0,23	0,51	68,07	79,94	55,28
SERPINF2	Alpha-2-antiplasmin	0,91	0,01	0,66	0,06	0,25	0,46	17,27	20,47	32,86
SERPING1	Plasma protease C1 inhibitor	-0,4	0,13	-0,47	0,07	0,07	0,78	447,95	470,1	350,15
SERPINH1	Serpin H1	-0,11	0,63	-0,08	0,74	-0,04	0,87	91,84	90,99	88,75
SERPINI1	Neuroserpin	-0,83	0,11	-0,48	0,35	-0,35	0,45	4,38	3,45	2,42
SERPINI2	Serpin I2	-0,63	0,49	-1,07	0,23	0,44	0,59	1,12	1,51	0,69

SPINK1	Serine protease inhibitor Kazal-type 1	-0,33	0,74	-1,25	0,19	0,92	0,31	3,15	5,92	2,63
SPINK5	Serine protease inhibitor Kazal-type 5	0,26	0,48	0,18	0,63	0,09	0,8	36,72	41,23	48,1
